# Large Cell Neuroendocrine Carcinoma of the Lung: Current Understanding and Challenges

**DOI:** 10.3390/jcm11051461

**Published:** 2022-03-07

**Authors:** Elisa Andrini, Paola Valeria Marchese, Dario De Biase, Cristina Mosconi, Giambattista Siepe, Francesco Panzuto, Andrea Ardizzoni, Davide Campana, Giuseppe Lamberti

**Affiliations:** 1 Department of Experimental, Diagnostic and Specialty Medicine, Sant’Orsola-Malpighi University Hospital, ENETS Center of Excellence, 40138 Bologna, Italy; elisa.andrini2@studio.unibo.it (E.A.); paola.marchese7@studio.unibo.it (P.V.M.); andrea.arddizoni2@unibo.it (A.A.); giuseppe.lamberti8@unibo.it (G.L.); 2 Division of Medical Oncology, IRCCS Azienda Ospedaliero-Universitaria di Bologna, Via P. Albertoni 15, 40138 Bologna, Italy; 3 Department of Pharmacy and Biotechnology, Molecular Diagnostic Unit, University of Bologna, Viale Ercolani 4/2, 40138 Bologna, Italy; dario.debiase@unibo.it; 4 Department of Radiology, IRCCS Azienda Ospedaliero-Universitaria di Bologna, University of Bologna, 40138 Bologna, Italy; cristina.mosconi@aosp.bo.it; 5 Radiation Oncology, IRCCS Azienda Ospedaliero-Universitaria di Bologna, 40138 Bologna, Italy; giambattista.siepe@aosp.bo.it; 6 Digestive Disease Unit, ENETS Center of Excellence of Rome, Sant’Andrea University Hospital, 00189 Rome, Italy; francesco.panzuto@uniroma1.it; 7 Department of Medical-Surgical Sciences and Translational Medicine, Sapienza University of Rome, 00189 Rome, Italy

**Keywords:** LCNEC, ICIs, next-generation sequencing, RB1, TP53, targeted therapies

## Abstract

Large cell neuroendocrine carcinoma of the lung (LCNEC) is a rare and highly aggressive type of lung cancer, with a complex biology that shares similarities with both small-cell lung cancer (SCLC) and non-small-cell lung cancer (NSCLC). The prognosis of LCNEC is poor, with a median overall survival of 8–12 months. The diagnosis of LCNEC requires the identification of neuroendocrine morphology and the expression of at least one of the neuroendocrine markers (chromogranin A, synaptophysin or CD56). In the last few years, the introduction of next-generation sequencing allowed the identification of molecular subtypes of LCNEC, with prognostic and potential therapeutic implications: one subtype is similar to SCLC (SCLC-like), while the other is similar to NSCLC (NSCLC-like). Because of LCNEC rarity, most evidence comes from small retrospective studies and treatment strategies that are extrapolated from those adopted in patients with SCLC and NSCLC. Nevertheless, limited but promising data about targeted therapies and immune checkpoint inhibitors in patients with LCNEC are emerging. LCNEC clinical management is still controversial and standardized treatment strategies are currently lacking. The aim of this manuscript is to review clinical and molecular data about LCNEC to better understand the optimal management and the potential prognostic and therapeutic implications of molecular subtypes.

## 1. Introduction

Lung neuroendocrine tumors account for approximately 20% of all lung cancers [1]. Pulmonary large-cell neuroendocrine carcinoma (LCNEC) is a rare but highly aggressive tumor with neuroendocrine differentiation, representing about 3% of all lung cancers [2,3]. LCNEC was first identified as a distinct subtype of lung cancer by Travis et al. and was then classified by the World Health Organization (WHO) as a subtype of large-cell carcinoma (LCC), i.e., a neuroendocrine non-small-cell lung cancer (NSCLC) [4,5,6]. However, in 2015, the WHO classification grouped lung neuroendocrine neoplasms (NENs) into one category including four major subtypes and with distinct prognosis: typical carcinoid (TC) and atypical carcinoid (AC), which represents low- and intermediate-grade neuroendocrine tumors; small-cell lung cancer (SCLC) and LCNEC, which instead represent high-grade neuroendocrine carcinomas [7]. The close relationship between SCLC and LCNEC is based on common clinicopathologic characteristics, such as aggressive behavior, strong association with smoking, higher incidence in males, high proliferation rate, neuroendocrine gene expression and poor prognosis [8]. Indeed, patients with LCNEC and SCLC are typically heavy smokers, with a median age of 65 years and metastatic disease at diagnosis, as opposed to carcinoids patients who are generally younger, have no clear association with smoking and have a better prognosis.

Typically, LCNEC and SCLC are characterized by higher mitotic rates and more extensive necrosis compared to lower grade neuroendocrine tumors, namely TC and AC. Both LCNEC and SCLC are highly aggressive tumors, with 5-year overall survival (OS) rates at advanced stage of approximately 15–25% and 5%, respectively [9,10]. Recently, the incidence of LCNEC has discreetly raised (from 0.01/100.000 in 1990 to 1.8/100.000 in 2010), probably due to the increased use of immunohistochemical neuroendocrine markers as diagnostic tools and to the greater awareness of LCNEC among pathologists [9,11].

Considering LCNEC rarity and clinicopathological similarities with both SCLC and NSCLC of lung cancer, treatment approaches of LCNEC are extrapolated from those of SCLC and NSCLC, since studies specifically addressing patients with LCNEC are small in size and retrospective in design [12,13]. Moreover, the characteristic heterogeneity of LCNEC, with some tumors carrying typical mutations of SCLC with low expression of neuroendocrine markers and other tumors exhibiting classical NSCLC mutations with a neuroendocrine expression profile, results in a lack of consensus in their clinical management according to SCLC-directed strategies as opposed to NSCLC-directed ones [10].

Furthermore, genomic and transcriptomic analyses have shown the existence of different molecular subtypes of LCNEC, which could potentially allow patients to receive more personalized treatments [10,14,15,16].

Clinical management of LCNEC is currently controversial and treatment strategies are not standardized due to the lack of definitive evidence supporting a specific approach, so that there is an emerging need to define the optimal management for this aggressive disease. The aim of this review is to collect current clinical and emerging molecular data regarding LCNEC, and to highlight new potential treatment strategies, also based on new molecular subtypes.

## 2. Pathological Diagnosis and Molecular Features

### 2.1. Morphology and Neuroendocrine Features

Macroscopically, most LCNECs appear as peripherally located nodular and necrotic masses of the lung. Histological/Cytological diagnosis of LCNEC is complex because small biopsies are usually insufficient due to crushing artifacts, whereas a surgical lung biopsy is usually required [17].

LCNEC appears as a high-grade malignant tumor with neuroendocrine morphology (organoid or trabecular patterns, rosette-like structures, or peripheral palisading), a non-small-cell cytology (with a cell size three times larger than lymphocytes diameter), abundant cytoplasm (often eosinophilic), prominent nucleoli and low nuclear-to-cytoplasmatic ratio, with frequent necrosis and a high mitotic rate, greater than 10 mitoses per 2 mm^2^ (average 60–80 mitoses per 2 mm^2^) [5,17,18,19].

Diagnosis of LCNEC is based on the identification of neuroendocrine morphology and the expression of at least one of the neuroendocrine markers (neural cell adhesion molecule [NCAM]/CD56, chromogranin A [CgA], synaptophysin) by immunohistochemical (IHC) staining or electron microscopy (almost supplanted by IHC). These are difficult to perform on small biopsies or cytology smears due to the typical crushing artifacts [20]. The most sensitive neuroendocrine biomarker is NCAM/CD56 (expressed in 92–98% of cases) but it has low specificity since it is also expressed in 10% of lung adenocarcinomas (LUADs), lung squamous cell carcinomas (LSCCs) and non-neuroendocrine large-cell carcinomas. CgA, expressed in approximately 70% of LCNECs, is the most specific marker but has low sensitivity. Synaptophysin, instead, is expressed in 87% of LCNECs but also in 10% of LUADs and 5% of LSCCs [10,21]. Moreover, thyroid transcription factor-1 (TTF-1) is positive in approximately 40–50% of cases compared to 85–90% of cases in SCLC.

Clinicopathological and molecular features of LCNEC as compared to NSCLC and SCLC are reported in Table 1. In equivocal cases, evaluation of the expression of the Retinoblastoma gene-1 (*RB1*) could be helpful, as loss of *RB1* expression is found in greater than 95% of SCLCs but only in in approximately 50% of LCNECs [14,22].

Recently, a common grading system for all thoracic NENs has been proposed by WHO and the International Association for the Study of Lung Cancer (IASLC), which includes the assessment of mitotic rate, expressed as mitoses per 10 high-power field (HPF) or 2 mm^2^, and the presence and extent of necrosis to differentiate low-, intermediate-, and high-grade NENs of the lung (Table 2) [27,28]. According to the proposed classification, well-differentiated NENs are also referred to as carcinoid tumors and include both low- and intermediate-grade tumors. Poorly differentiated NENs include SCLC and LCNEC [27]. Moreover, according to the WHO classification, LCNEC can be divided into pure LCNEC and combined LCNEC, the latter mixed with components of adenocarcinoma, squamous cell carcinoma, and other rare subtypes (e.g., spindle-cell carcinoma or giant cell carcinoma). LCNEC combined with SCLC must include at least a 10% component of LCNEC [10,17]. The Ki67 proliferation index might help in characterizing the combined LCNEC and defining prognosis [29].

### 2.2. Molecular Characterization and Subtyping

LCNEC shows a high gene mutation transversion:transition ratio which is associated with smoking and a high tumor mutational burden (8.5 to 10.5 mutations per megabase) [14,15]. Moreover, the widespread availability of next-generation sequencing (NGS) techniques allowed genomic studies to be performed on LCNEC and the identification of at least two distinct molecular subtypes of LCNEC.

Rekhtman and colleagues performed NGS of 45 histologically pure LCNECs and identified two major subsets: a SCLC-like subset (40%) with concurrent alteration of tumor protein p53 (*TP53*) and *RB1*, and an NSCLC-like subset (56%), lacking *RB1* and *TP53* co-alteration and characterized by NSCLC typical mutations such as serine/threonine kinase 11 (*STK11*), Kirsten rat sarcoma viral oncogene homolog (*KRAS*) mutations (characteristic of LUAD), and kelch-like ECH associated protein 1 (*KEAP1*) mutations (typical of both LUAD and LSCC) [14,23,24,30]. In addition, a third subset of carcinoid-like LCNEC (4%) was identified which lacked *RB1* and *TP53* alterations and was characterized by low mutational burden and *MEN1* alterations, hallmarks of carcinoids. Interestingly, *STK11* mutations were more frequent in NSCLC-like LCNEC compared to lung LUAD (60% vs. 16%, respectively), suggesting that *STK11* mutations, which are associated with rapid tumor growth and metastasis in lung LUAD, can explain the aggressive clinical behavior of LCNEC, as observed in NSCLC [31]. Furthermore, the SCLC-like subset harbored exclusive alterations of SCLC, e.g., v-myc avian myelocytomatosis viral oncogene lung carcinoma derived homolog gene *(MYCL)* amplification, sex-determining region Y (SRY)-box 2 (*SOX2*) amplification, phosphatase and tensin homolog (*PTEN*) mutation/loss and Fibroblast Growth Factor Receptor 1 (*FGFR1*) amplification, with complete absence of *STK11* and *KRAS* mutations, whereas the NSCLC-like subset occasionally showed SCLC typical alterations (e.g., *MYCN* amplification). Finally, this study highlighted the higher mutation burden of both SCLC-like and NSCLC-like subsets compared to conventional NSCLC and SCLC, suggesting that LCNEC could be sensitive to immune checkpoint inhibitors (ICIs) [32].

Similarly, George and colleagues performed a comprehensive molecular analysis of 75 pulmonary LCNECs (19 with combined histology), identifying two distinct genomic subgroups with specific transcriptional patterns: type I LCNEC (37%), characterized by *TP53* and *STK11/KEAP1* alterations, similar to lung adenocarcinoma and squamous cell carcinoma [23,24,30], and type II LCNEC (42%), with concurrent inactivation of *TP53* and *RB1*, typical of SCLC [15]. Regarding transcriptional patterns, type I LCNECs showed a neuroendocrine expression profile, with high expression of Achaete-scute homolog 1 (*ASCL1*) and Delta-like ligand 3 (*DLL3*) genes and downregulation of NOTCH pathway genes (*ASCL1^high^/DLL3^high^/NOTCH^low^*), whereas type II LCNECs exhibited low expression of neuroendocrine markers, *ASCL1* and *DLL3, and NOTCH* upregulation (*ASCL1^low^/ DLL3^low^/NOTCH^high^*). Interestingly, type II LCNECs also showed an upregulation of immune-related pathways, with possible implications with respect to response to ICIs [15]. Based on these data, type I LCNEC has a mutational pattern similar to NSCLC but gene expression profiles typical of SCLC, whereas type II LCNEC has a mutational pattern of SCLC but low expression of neuroendocrine markers, similar to NSCLC.

Miyoshi and colleagues performed a genomic profiling analysis of 78 LCNEC samples (including 65 surgically resected cases and 13 advanced cases) and compared the genomic profiles of LCNECs with those of 141 SCLCs (including 50 surgically resected cases and 91 advanced cases) [33]. The study showed similar genomic profiles between LCNEC and SCLC, including the high frequency of inactivating mutations in *TP53* and *RB1*, with a significantly lower prevalence of *RB1* mutations in LCNEC compared to SCLC (40%), and genetic alterations in the PI3K/AKT/mTOR pathway, which could represent potential therapeutic targets. Moreover, LCNEC harbored other potentially targetable activating alterations in *FGFR1* (5%), Erb-B2 Receptor Tyrosine Kinase 2 (*ERBB2*) (4%) and Epidermal Growth Factor Receptor (*EGFR*) (1%). Interestingly, among 10 cases of combined LCNECs with NSCLCs, the study detected highly concordant genetic alterations, including driver alterations, between the two components, with a median concordance rate of 71%. These observations suggest a common precursor cell for two components (LCNEC and NSCLC) and that agents exploiting these mutations could be active against both components.

As the same gene alterations typical of high-grade tumors were identified with a lower frequency also in low-grade ones, some studies suggested that high-grade neuroendocrine carcinomas could develop from preexisting carcinoids [34,35]. A comparative transcriptomic analysis of ACs and LCNECs identified three transcriptional clusters with specific genomic patterns: cluster 1 (LCNEC-enriched group), including 20 LCNECs and 1 AC, showed concurrent inactivation of *TP53* and *RB1* (100%) and frequent alterations in *SMARCA2* (19%), *STK11*, *KEAP1* and *MYCL1* (each 14.3%); cluster 3 (AC-enriched group), comprising 20 ACs and 4 LCNECS, characterized by frequent *MEN1* (37.5%) and *TP53* (16.7%) mutations, without *RB1* alterations; cluster 2, including 14 ACs and 8 LCNECs, showed intermediate features, with frequent *TP53* alterations (40.9%) followed by *MEN1* (22.7%) and *RB1* (18.2%) [36]. These clusters showed some interesting overlap with the molecular subsets defined by Rekhtman and George. The LCNEC-enriched cluster 1 showed similar molecular features to SCLC-like LCNEC subset of the study by Rekhtman et al. and to the type II NSCLC-like LCNEC from the study by George et al., whereas cluster 2 showed similarities with the NSCLC-like LCNEC subset from the study by Rekhtman et al. and to the type I from the study by George et al.

Finally, ACs in cluster 2 may overlap with the recently identified “supracarcinoids”, a subgroup of lung NENs with a carcinoid-like morphology but with molecular features typical of LCNEC [37]. These data support the hypothesis of a progression of malignancy from carcinoids to high-grade neuroendocrine carcinomas through the accumulation of gene alterations. Whether supracarcinoids should be treated as ACs or as LCNECs is currently unknown. Interestingly, circulating cell-free DNA (cfDNA) sequencing could be a reliable alternative for genomic subtyping of LCNEC as it showed a 90% concordance with tumor DNA (see also below “Treatment” section) [38]. Considering the prognostic and potential therapeutic implications, genetic characterization should be performed in advanced LCNEC patients, whenever possible, to understand the nature of LCNEC.

## 3. Staging

On a diagnostic computed tomography (CT) scan, LCNEC often appears as a peripherally located lesion, with well-defined (64%), lobulated (93%) or, less frequently, spiculated (41%) margins, and calcifications in 10% of cases [20,39,40].

Given the low differentiation and high expression of the glucose transporter 1 (GLUT1), LCNEC cells are 18F-fluorodeoxyglucose ([18F]-FDG) avid, thus making [18F]-FDG positron emission tomography ([18F]-FDG-PET) a useful staging tool with high sensitivity and specificity and consequently a valid imaging assessment to select patients with early-stage disease for radical treatment [41]. Biopsy through bronchoscopy (possibly with endoscopic ultrasonography) is recommended for both diagnosis and staging of locally advanced tumors, but for a correct pathological diagnosis a surgical specimen would be needed (see “Pathology” section) [41]. The IASCL has suggested the application of the tumor—node—metastasis (TNM, 8th revision) staging to all NENs of the lung. However, this staging system is not accurate in predicting the prognosis of patients with LCNEC, because it does not include metastasis sites/patterns, pathological and genetic factors that are associated with different prognosis, so that a more comprehensive classification that includes these factors is needed [42]. The identification of localized, compared to locally advanced and metastatic disease, is crucial for treatment planning.

## 4. Prognostic Factors

The prognosis of patients with LNCEC is dismal overall, with a five-year survival rate as low as 8% in the advanced stage [43]. Age, sex, primary tumor size, lymph node metastases, and stage were identified as prognostic factors for both survival (OS and survival rates at three and five years), and lung cancer-specific survival in population-based studies [44,45]. In addition, surgery of the primary tumor was independently associated with survival [44]. Moreover, a recent multi-institutional retrospective study investigated outcomes of 251 patients with LCNEC after surgical resection and showed that only lymphatic invasion was an independent prognostic factor [46]. The simultaneous expression of at least two neuroendocrine markers at IHC was associated with worse survival after surgical resection and adjuvant chemotherapy, as reported by the aforementioned study [46] and according to a series of 63 patients with resected LCNEC [47]. Indeed, patients with non-triple-positive LCNEC (i.e., tumors not immunoreactive to all three neuroendocrine markers) had a significantly higher five-year survival rate compared to triple-positive ones.

The newly identified molecular subtypes of LCNEC currently lack a clear prognostic correlate: indeed, SCLC-like LCNEC was associated with a not significant trend toward shorter RFS compared to the NSCLC-like one, with no difference in terms of OS [14].

These data suggest that lymphatic invasion and the presence of neuroendocrine IHC markers might be potentially considered as prognostic factors in an integrated LCNEC-specific staging system [48,49].

## 5. Treatment

### 5.1. Early-Stage Disease

LCNEC is diagnosed at an early stage (i.e., I-IIIA according to AJCC 7th edition) in about 25% of cases and, despite the lack of strong evidence, surgery is considered the cornerstone of treatment in patients with early-stage LCNEC in those fit for surgery [12,50,51]. According to stage, patients who undergo surgery achieve five-year survival rates of 27–67% in stage I, 18–75% in stage II and 8–45% in stage III [52,53,54]. The high variability of reported survival rates likely reflects the high heterogeneity of LCNEC, which could influence survival, in addition to tumor stage.

However, retrospective and population-based studies have reported that surgery yields a significant survival improvement in patients with early stage LCNEC and that it was independently associated with better OS [50,55,56]. In respect to the type of surgery, an analysis of data from the Surveillance, Epidemiology, and End Results (SEER) database including 1530 patients with any-stage LCNEC showed that the lobectomy/bilobectomy approach was associated with better outcomes when compared to pneumonectomy or wedge resection/segmentectomy [55]. Furthermore, multimodal treatment (surgery with chemotherapy and/or radiotherapy) was associated with better prognosis compared to surgery alone, in particular the combination of surgery and chemotherapy showed a significant survival improvement compared to other types of treatments [56].

Considering the high recurrence rate after surgery (64% within one year, 91% within three years) [53], perioperative treatments with neoadjuvant and/or adjuvant chemotherapy could improve survival, even for node-negative disease [57,58].

The rarity of LCNEC makes it difficult to perform prospective studies and thus to properly evaluate the real value of adjuvant chemotherapy, but several retrospective studies showed a probable benefit of cisplatin-based regimens in this setting [52,54,59,60]. Response rates of about 80%, comparable to that observed in patients with SCLC, have been reported with neoadjuvant chemotherapy [61,62]. Two large population-based analyses of data about patients with stage I LCNEC from the National Cancer DataBase (NCDB) showed that adjuvant chemotherapy was beneficial in stage IB tumors, while data about stage IA are controversial [59,63]. Accordingly, a retrospective study of 1770 patients with resected early-stage, node-negative LCNEC showed that adjuvant chemotherapy was associated with better OS, especially if tumor size was greater than 3 cm, and if chemotherapy was administered within three to six months after surgery, whereas no advantage was observed if the tumor was smaller than 2 cm and if chemotherapy was started after six months post-surgery [64]. In these studies, five-year survival rates were 60–65% for patients who received chemotherapy and 42–48% for those who did not [59,64]. To better select patients with LCNEC who could benefit from adjuvant chemotherapy, a retrospective study evaluated 63 patients with surgically-resected LCNEC and showed that perioperative chemotherapy was associated with better OS compared to surgery alone, particularly in the non-triple-positive group (i.e., tumors not immunoreactive to all three neuroendocrine markers) [47]. Moreover, non-triple-positive patients who underwent surgery with perioperative chemotherapy had a significantly greater five-year survival compared to those who underwent surgery alone. No difference was found in the triple-positive group. Also, a reduced benefit from adjuvant chemotherapy on OS was observed for CgA-positive (at IHC) tumors.

A small single-arm prospective trial of adjuvant chemotherapy with cisplatin-etoposide in 15 resected LCNEC patients showed a significant survival improvement in these patients when compared to historical data (five-years OS of 88.9% vs. 47.4%, respectively) [65].

With respect to the type of adjuvant chemotherapy, a phase III trial enrolling patients with high-grade NENs of the lung (both LCNEC and SCLC) failed to demonstrate the superiority of cisplatin-irinotecan compared to cisplatin-etoposide as an adjuvant regimen [66]. Indeed, the trial was stopped prematurely due to futility: at a median follow-up of 24.1 months, the 3-year relapse-free survival rate was not different between the two arms, being 65.4% for the cisplatin plus etoposide arm compared to 69% for the cisplatin plus irinotecan one [67].

The role of radiotherapy (RT) in the treatment of pulmonary LCNEC is still unclear, but some authors suggest its use in the setting of non-metastatic disease [68]. A large retrospective study compared survival outcomes among stage I-IIIA LCNEC patients from the NCDB who received definitive chemoradiation or surgery [69]. The study showed a significative survival benefit with surgery among 3371 stage I LCNEC compared to stereotactic body RT (SBRT) (five-years OS 50% versus 27%) and among stage II (N = 1150) and IIIA (N = 1437) patients compared to definitive chemoradiation (five-years OS 45% vs. 12%; 36% vs. 25%, respectively). Another retrospective analysis compared SBRT with surgery in 3344 patients with stage I LCNEC, showing a median OS (mOS) of 34.6 months with SBRT compared to 57.2 months with surgery and a five-year OS of 25% vs. 48% [70]. These results must be considered carefully because registry-based studies are subjected to bias, since surgery is often offered and performed in patients in better general conditions as compared to less invasive approaches, such as SBRT.

Furthermore, the role of post-operative RT was evaluated in two large retrospective trials among patients with resected early-stage LCNEC, demonstrating that RT was not associated with survival benefit [63,64]. On the contrary, a recent large-population based study including 1480 LCNEC patients showed that RT could yield a survival benefit in patients with stage II-III LCNEC, a tumor size of 5–10 cm, and who had not been treated with surgery or were among patients who received chemotherapy [71].

To sum up, dedicated guidelines to support the management of patients with early-stage LCNEC are lacking. Nevertheless, available data suggest that surgery is currently the mainstay of treatment in patients with resectable LCNEC, whereas RT should be preferentially reserved for patients not eligible for surgery, particularly in the context of a multimodal approach that includes chemotherapy. The reviewed studies are reported in Table 3.

### 5.2. Locally-Advanced Disease

Locally-advanced LCNEC, namely stage IIIB-C, is diagnosed in approximately 20% of cases [72,73]. Current practice regarding the management of patients with stage III LCNEC is derived from retrospective trials or from studies conducted in patients with NSCLC/SCLC. Many retrospective studies suggest that the ORR of advanced LCNEC to cisplatin-based chemotherapy was comparable to that of SCLC [74,75]. Some authors also suggest a role for RT in this setting [68].

A large retrospective study of 5797 patients with locally-advanced LCNEC compared the use of definitive chemoradiotherapy to chemotherapy alone, showing an mOS of 16.1 months in the chemoradiotherapy group compared to 11.9 months in the chemotherapy group [76]. Similar results were reported by another study where patients with unresectable LCNEC (including stage I and II) treated with chemoradiotherapy had better outcomes in terms of OS compared to those treated with chemotherapy alone [56].

Considering the limited data and the absence of currently available large prospective clinical trials, chemoradiotherapy with platinum-etoposide for four cycles could represent an effective treatment for patients with unresectable stage III LCNEC.

Prophylactic cranial irradiation (PCI) in limited-stage SCLC with complete remission after chemoradiotherapy is associated with a lower risk of symptomatic brain metastases and improved OS [77]. Similar data has been observed in locally advanced NSCLC not progressing after treatment. PCI improved brain metastases rate and RFS, but not OS [78]. In patients with LCNEC, an Italian retrospective study of 72 patients with stage III-IV LCNEC showed that PCI (with a total dose of 25 Gy in 10 fractions) was associated with a trend toward an increased mPFS (20.5 versus 6.4 months, *p* = 0.09) and mOS (33.4 versus 8.6 months, *p* = 0.05) [79], and can thus be considered in this setting.

### 5.3. Advanced Disease

Most of the patients with LCNEC (40–50%) are diagnosed with metastatic disease [46,72]. First-line treatment of metastatic LCNEC is still debated since it is an overall rare disease that shares clinical and molecular characteristic with both SCLC and NSCLC. Indeed, most of the data about stage IV LCNEC are extrapolated from SCLC and NSCLC practice, because specific studies are small and retrospective and large prospective clinical trials are lacking [74,80,81,82]. However, many studies supported the efficacy of drugs usually given in SCLC, i.e., platinum-etoposide (SCLC-like regimen), for patients with advanced LCNEC, as opposite to those commonly used in NSCLC, namely platinum-pemetrexed/gemcitabine/taxane (NSCLC-like regimens) [81,83,84].

The only prospective evidence comes from a phase II multicenter study which evaluated the efficacy of cisplatin-etoposide among 42 patients with untreated advanced LCNEC [81]. The median progression-free survival (mPFS) was 5.2 months, whereas mOS was 7.7 months, slightly worse than what was reported for patients with SCLC treated with the same drugs. A retrospective study of 83 cases of patients with any-stage LCNEC demonstrated that, also in a metastatic setting, platinum-etoposide chemotherapy, as compared to NSCLC-based regimens, was associated with better mOS (51 vs. 21 months, respectively) and with an ORR of 29%, with complete or partial response only in patients receiving SCLC-based regimens [85]. A large series of 294 metastatic LCNEC patients compared the efficacy of different chemotherapy regimens grouping patients as follows: group I (NSCLC-like chemotherapy), which included patients treated with gemcitabine, docetaxel, paclitaxel or vinorelbine; group II (pemetrexed NSCLC type) which included patients treated with pemetrexed-containing regimens; and group III (SCLC-like type), which included patients who received etoposide-based chemotherapy. Patients with LCNEC in group I showed a longer OS than those in group II and III, with mOS of 8.5, 5.9, and 6.7 months, respectively [83]. Another retrospective study compared the efficacy of SCLC-like chemotherapy with NSCLC-like regimens in 45 advanced LCNEC patients [13]. Outcomes of the SCLC-like regimen (N = 11) were better compared to NSCLC-like ones (N = 34), with an ORR of 73% and 50%, an mPFS of 6.1 months and 4.9 months, and a mOS of 16.5 and 9.2 months, respectively.

Efficacy of different chemotherapy drugs may vary depending on the genomic profile of the tumor and cfDNA analysis can be a useful and convenient way to subgroup LCNEC and drive treatment.

In a retrospective study, 63 patients with LCNEC were divided according to cfDNA and tumor DNA profile in SCLC-like (loss of both *RB1* and *TP53*) and NSCLC-like subtypes [38]. The SCLC-like group had overall shorter OS and higher ORR to chemotherapy as compared to the NSCLC-like one. However, patients in the SCLC-like group showed higher ORR and longer OS on platinum-etoposide chemotherapy (SCLC-like regimen) as compared to platinum-pemetrexed or platinum-gemcitabine/taxane (NSCLC-like regimens preferentially used in adenocarcinoma and squamous histology, respectively). Contrary to this, patients in the NSCLC-like group had shorter OS on platinum-gemcitabine/taxane chemotherapy as compared to platinum-etoposide and platinum-pemetrexed. Accordingly, patients with *RB1*-wild-type advanced LCNEC had a significantly longer OS when treated with NSCLC-based chemotherapy (i.e., platinum-gemcitabine/taxane) compared with platinum-etoposide chemotherapy (9.6 months vs. 5.8 months, respectively), while no difference was observed in *RB1* mutated patients treated with different chemotherapy regimens [84]. In contrast to previously reported data, a retrospective study evaluated 49 stage IV LCNEC patients, of whom 70% (N = 26) received platinum-etoposide as a first-line treatment and 30% (N = 11) received other regimens; it showed worse OS among patients who received platinum-etoposide compared to those treated with other regimens (mOS 8.3 months vs. 19.5 months) [19]. However, considering all available evidence, four to six cycles of cisplatin or carboplatin combined with etoposide are generally administered in patients with advanced LCNEC, but the prognosis remains poor, with a mOS of approximately 8–12 months [57,81].

Since advanced LCNEC invariably progresses to first-line treatment, the efficacy of other chemotherapy regimens has been investigated in these patients. Different chemotherapy regimens (platinum-based combination regimens or monotherapy with irinotecan or vinorelbine or docetaxel) might be active in LCNEC, as shown by a retrospective study in patients with unresectable LCNEC that have been compared to a cohort of patients with SCLC. The study reported similar outcomes in the two groups of patients with ORR of 50% for patients with LCNEC and 53% in patients with SCLC and mOS of 10 and 12.3 months, respectively [82]. Furthermore, irinotecan and paclitaxel, drugs commonly used as second-line treatment in SCLC, with or without platinum, showed some degree of activity in 22 patients with advanced LCNEC, with an ORR of 70%, a mOS of 10.3 months and a one-year survival rate of approximately 40% for both drugs [86]. Similarly, in a previously mentioned study, taxanes or irinotecan (N = 11) showed a greater efficacy as compared to chemotherapy regimens commonly used in NSCLC (pemetrexed, gefitinib or erlotinib, N = 34) [13]. A phase II study investigated the efficacy of the combination of irinotecan with cisplatin in patients with advanced LCNEC (N = 30) as compared to patients with SCLC (N = 10). The ORR was 40% versus 80% and mOS 12.6 versus 17.3 months for patients with LCNEC and SCLC, respectively, showing that this regimen was overall less active in LCNEC than in SCLC [87].

Everolimus is a tyrosine-kinase inhibitor (TKI) of mTOR. A multicenter phase II trial evaluated the efficacy of everolimus in combination with paclitaxel and carboplatin for four cycles followed by maintenance everolimus until progression in 49 chemotherapy-naïve patients with stage IV LCNEC [88]. The ORR was 45%, the disease control rate was 74%, the mPFS was 4.4 months and the mOS was 9.9 months, demonstrating that this combination may be active as a first-line treatment in metastatic LCNEC. The combination was well-tolerated, with most frequent observed all-grade adverse events (AEs) being fatigue (22%), diarrhea (22%), anemia (20%), neutropenia (18%) and alopecia (18%), as expected. Everolimus-related AEs (i.e., stomatitis and rash) occurred in a minority of patients.

First-line treatment of metastatic LCNEC is still controversial, since most of the available studies are small and retrospective and some also showed conflicting results. However, platinum-etoposide chemotherapy seems to be more active than other regimens commonly used in NSCLC, but larger and prospective studies are needed to define the optimal treatment in this setting and the predictive value of molecular alterations, such as *TP53* and *RB1* status. Reviewed studies are reported in Table 4.

## 6. Future Perspectives

### 6.1. Targeted Therapies

While they are seldom detected in *pure* LCNEC, targetable driver mutations can occur in mixed forms of LCNEC-adenocarcinoma, especially *EGFR* mutations [10]. Molecular targeted therapy is based on the phenomenon of oncogene addiction and currently represents the cornerstone for treatment of oncogene-addicted NSCLC [89]. The first case of LCNEC harboring *EGFR* mutation treated with a EGFR-targeting TKI was reported about 10 years ago: a 66-year-old, never-smoker female was diagnosed with stage IV LCNEC carrying an *EGFR* exon 19 deletion and received gefitinib, a first-generation EGFR inhibitor, showing a dramatic response after two months of treatment, with a time to progression longer than six months [90]. Another case of metastatic LCNEC harboring an *EGFR* exon 19 deletion and treated with icotinib, another first-generation EGFR inhibitor, exhibited a long-lasting response for eight months [91]. A few cases of patients whose LCNEC harbored anaplastic lymphoma kinase (*ALK*)-translocation, which is another driver alteration commonly found in young, non-smoker patients with NSCLC and treated with selective TKI were reported, with controversial results [92,93].

Alterations of the PI3K/AKT/mTOR pathway are commonly encountered in SCLC and LCNEC [33]. A Japanese study genomically profiled SCLC and LCNEC samples, reporting changes in the PI3K/AKT/mTOR pathway in *PI3KCA* (3%), *PTEN* (4%), *AKT2* (4%) *RICTOR* (5%), *mTOR* (1%) and alterations in *EGFR* (1%), *ERBB2* (4%) and *FGFR1* (5%) in the LCNEC group, with similar frequency to what was observed in the SCLC group. Apart from the aforementioned study of everolimus, an mTOR inhibitor [88], no drugs exploiting PI3K/AKT/mTOR pathway activation or other of the mutations harbored by LCNEC have been successfully adopted to date. High expression rates of Vascular-Endothelial Growth Factor (VEGF), Human Epidermal Growth Factor Receptor 2 (HER 2) and c-KIT were found in LCNEC, suggesting a possible role of therapies targeting these alterations, e.g., antiangiogenics, HER-2 inhibitors such as trastuzumab, and KIT inhibitors such as imatinib [94].

Because prospective studies are difficult to carry on in such a rare and controversial disease, international efforts would be needed, hopefully in modern-designed trials, such as umbrella trials, to investigate the activity and efficacy of targeted therapies in patients with LCNEC. To date, considering the limited data on the efficacy of targeted therapy in LCNEC, NGS is not routinely recommended but could be useful after progression to first-line treatment or in non-smoker patients.

### 6.2. Immunotherapy in LCNEC

Recently, the combination of immunotherapy with platinum-etoposide chemotherapy has been established as the standard first-line treatment in advanced SCLC based on the results of two randomized large prospective trials demonstrating a significative OS benefit compared to chemotherapy alone [95,96]. However, to date, the efficacy of immunotherapy in LCNEC has not yet been established due to the rarity of this disease and to the consequent lack of prospective evidence. Most data derive from small and retrospective case series and case reports. A small case series including 10 patients with advanced LCNEC treated with single-agent nivolumab or pembrolizumab after progression to platinum-based first-line showed an ORR of 60% with a mPFS of about 14 months [97]. A recent retrospective study evaluated the activity and safety of ICIs in 37 patients with advanced LCNEC who were divided into two groups: group A1 (N = 23) treated with immunotherapy as a monotherapy or as a combination of different ICIs, and group A2 (N = 14) who did not receive ICIs [98]. Patients in group A1 showed promising outcomes with an ORR of 33%, an mPFS of 4.2 months and a mOS of 11.8 months, similar to what was observed in ICI-treated patients with NSCLC. A prospective, open-label, multicenter phase II trial is currently investigating the efficacy and safety of ipilimumab plus nivolumab across multiple rare tumors, and recently the results of the non-pancreatic neuroendocrine cohort have been reported [99]. Of note, among 32 eligible patients, of whom 19% had lung high-grade neuroendocrine carcinoma, the ORR was 44%. Similarly, the NIPINEC study is a French phase II trial of nivolumab ± ipilimumab in patients with LCNEC or high-grade gastroenteropancreatic neuroendocrine tumors that has ORR as the primary endpoint and is currently recruiting patients (NCT03591731). In addition, a phase II multicenter single-arm trial of atezolizumab in pretreated advanced patients with NSCLC with rare histology subtypes (CHANCE trial) is currently ongoing and also includes patients with LCNEC [100]. In addition, an Italian multicenter, phase II, single-arm study investigating the efficacy and safety of durvalumab in combination with carboplatin plus etoposide for four cycles followed by durvalumab maintenance in metastatic LCNEC (DUPLE trial) is ongoing (Eudract Number: 2020-005942-41). Of note, a phase II trial is also investigating the activity of the combination of nivolumab and temozolomide (NCT03728361), an alkylating agent more effective in tumor cells with methylation of the promoter of the gene encoding the O6-methylguanine methyltransferase (MGMT), a DNA damage repair (DDR) enzyme, which is a predictive factor of response to temozolomide in neuroendocrine tumors [101]. Methylation of the MGMT promoter could also identity LCNEC with DDR deficiency, which might be associated with benefit to immunotherapy [102,103]. Ongoing clinical trials in advanced and metastatic LCNEC are reported in Table 5.

A retrospective analysis including 661 stage IV LCNEC patients from the NCDB evaluated the impact of ICIs on the OS of 37 patients. The use of ICIs was associated with improved OS, whereas 12 and 18-month survival rates were 34.0% and 29.1%, respectively, compared to 24.1% and 15.0% in the non-ICI group [104]. Furthermore, a real-world retrospective analysis investigated the outcomes of ICIs among 125 advanced LCNEC patients who were divided into two groups: group A (N = 41), who received ICI as any treatment line, and group B (N = 84), who did not receive ICI [105]. The study showed a positive impact of ICIs on OS, with a mOS of 12.4 months in patients who received ICI compared to 6.0 months in patients who did not. Of note, ICI administration, as well as chemotherapy administration, ECOG PS at diagnosis, and the presence of liver metastasis, were independently associated with OS. Another retrospective study evaluated the efficacy of nivolumab among 51 advanced LCNEC patients, including 17 treated with nivolumab as second-line treatment or beyond [106]. The mOS from the start of nivolumab was 12.1 months, the ORR was 29.4%, the DCR was 58.8% and the mPFS was 3.9 months, with a median duration of response of 6.5 months.

As recently observed, the non-neuroendocrine SCLC inflamed phenotype (SCLC-I) showed increased susceptibility to the addition of anti-programmed death ligand 1 (PD-L1) inhibition to chemotherapy [107], but to date no correlation between LCNEC molecular subtypes and outcome to ICIs has been observed. Because of the dramatic impact of immunotherapy with ICIs in other tumor types, e.g., NSCLC, melanoma and renal cell carcinoma, the investigation of this treatment strategy in LCNEC could be crucial to improving prognosis.

## 7. Conclusions

LCNEC is a rare and aggressive lung tumor with a dismal prognosis and a challenging management. Because of its rarity and the peculiar biology, which is still largely not defined, patients with LCNEC should be addressed to tertiary centers with specific knowledge about rare neuroendocrine lung disease for a careful evaluation and treatment. Treatment strategies in both early-stage and advanced disease are not yet established considering the lack of prospective evidence. However, in current clinical practice, patients with advanced LCNEC receive chemotherapy regimens commonly used for SCLC patients, with similar or worse results in terms of response and survival. Recent findings about LCNEC molecular subtypes suggest that the treatment approach could be driven by molecular features, but further and larger studies are needed. Moreover, limited but promising data were reported regarding the efficacy of ICIs and, to a lesser extent, targeted therapies in these patients. Therefore, molecular testing and the use of genetic signatures could represent important tools to better understand the nature of LCNEC and to select the best treatment approach in this rare disease. Finally, the inclusion of LCNEC patients in clinical trials is strongly recommended in order to validate new treatment options and potentially correlate genomic profiles with response to different treatment strategies.

## Figures and Tables

**Table 1:** Comparison of clinicopathological and molecular features of non-small-cell lung cancer (NSCLC), small-cell lung cancer (SCLC), and large-cell neuroendocrine carcinoma (LCNEC).

	NSCLC	SCLC	LCNEC
% of lung tumors	76%	15–20%	2–3%
Association with smoking	Variable	Strong	Strong
Histopathological features	LUAD: glandular differentiation or mucin production LSCC: squamous cell differentiation (i.e., keratinization, keratin pearl formation and intercellular bridges) with moderate to abundant cytoplasm	Dense proliferation of small tumor cells, scant cytoplasm, finely granular chromatin, inconspicuous nucleoli, nuclear molding, extensive necrosis, crushing artifacts	Cell size 3× lymphocytes diameter Abundant cytoplasm Prominent nucleoli Frequent necrosis
IHC	TTF-1 in LUAD (>85%) p40 in LSCC	TTF-1 (85–90%) Neuroendocrine markers (CgA, NCAM/CD56, Syn)	TTF-1 (40–50%) Variable expression of neuroendocrine markers (CgA, NCAM/CD56, Syn)
Location of primary tumor	LUAD: peripheral LSCC: central	Central	Peripheral
Molecular patterns	Oncogene-addicted (~30%) Six molecular subtypes in LUAD [23], four in LSCC [24] Non-oncogene addicted (~70%)	SCLC-A (*ASCL1*) SCLC-N (*NEUROD1*) SCLC-P (*POU2F3*) SCLC-Y/I (*YAP1*/Inflamed) [25]	Type I (*TP53, KEAP1, STK11*) Type II (*TP53* and *RB1* co-inactivation)
Sensitivity to chemotherapy and standard first-line	Variable Platinum-based plus pembrolizumab TKI in oncogene-addicted	High Platinum plus etoposide [26]	Variable NSCLC chemotherapy for type I SCLC chemotherapy for type II
Five-year survival rate	25%	7%	15–57%

NSCLC: non-small cell lung cancer; SCLC: small-cell lung cancer; LCNEC: Large-cell neuroendocrine carcinoma; IHC: Immunohistochemistry; TTF-1: thyroid transcription factor-1; LUAD: lung adenocarcinoma; LSCC: lung squamous cell carcinoma; CgA: Chromogranin A; NCAM: neural cell adhesion molecule; Syn: Synaptophisin; *ASCL1*: Achaete-scute homolog 1; *NEUROD1*: neurogenic differentiation factor 1; *POU2F3*: POU class 2 homeobox 3; *YAP1*: yes-associated protein 1; *TP53*: tumor protein p53; *RB1*: Retinoblastoma gene-1; *KEAP1*: kelch-like ECH associated protein 1; *STK11*: serine/threonine kinase 11; TKI: tyrosine-kinase inhibitor.

**Table 2:** Grading Systems for NETs of the lung and thymus adapted from IASLC 2018 and WHO 2021 [27,28].

*Grade*		*WHO, IASLC*	
*Low*	Typical Carcinoid	<2 mitoses/10 HPF, no necrosis	Neuroendocrine carcinoma, G1
*Intermediate*	Atypical carcinoid	2–10 mitoses/10 HPF, foci of necrosis	Neuroendocrine carcinoma, G2
*High*	Large-cell neuroendocrine carcinoma	9–10 mitoses/10 HPF	Neuroendocrine carcinoma, G3
	Small-cell carcinoma		

WHO: World Health Organization; IASCL: International association for the study of lung cancer; HPF: high-power field; G: grade.

**Table 3:** Summary of available studies evaluating treatment of early stage LCNEC.

Author	Type of Study	*n*. of Patients	Treatment	Results
Veronesi et al. (2006) [61]	Retrospective	144	Neoadjuvant chemotherapy vs. adjuvant chemotherapy	5 y OS of 42.5%
Kujtan et al. (2018) [59]	Population analysis (NCDB) Stage I	1232	Surgery combined with adjuvant chemotherapy (275) vs. surgery alone (957)	Adjuvant chemotherapy better in OS Five y OS of 64.5% vs. 48.4% Stage IA HR 0.64, 95% CI [0.47–0.88] Stage IB HR 0.43, 95% CI [0.32–0.59]
Raman et al. (2019) [63]	Population analysis (NCDB) Stage I	2641	Surgery combined with adjuvant chemotherapy (481) vs. surgery alone (2161)	Adjuvant chemotherapy better in OS mOS 81 vs. 65 m Stage IA HR 0.92, 95% CI [0.75–1.11] Stage IB HR 0.67, 95% CI [0.50–0.90]
Cao et al. (2019) [55]	Population analysis (SEER)	1530	Segmentectomy/wedge resection Lobectomy/Bilobectomy Pneumonectomy Chemotherapy Radiation	HR: 0.526, 95% CI [0.413–0.669] HR: 0.357, 95% CI [0.290–0.440] HR: 0.491, 95% CI [0.355–0.679] HR: 0.442, 95% CI [0.389–0.503] HR: 0.837, 95% CI [0.738–0.949]
Gu et al. (2019) [56]	Population analysis (SEER)	2594	Surgery combined with chemotherapy vs. surgery alone Surgery combined with chemotherapy vs. surgery with other treatments	*p* = 0.044 *p* = 0.033
Iyoda et al. (2006) [65]	Prospective (phase II, single arm)	50	cisplatin and etoposide vs. retrospective arm (surgery alone)	Adjuvant chemotherapy better in OS Five y OS of 88.9% vs. 47.4% (*p* = 0.0252)
Kenmotsu et al. (2020) [67]	Prospective (phase III, two arms)	221	Cisplatin + Irinotecan vs. Cisplatin + Etoposide	Three y RFS 69% vs. 65%, 95% CI [0.66–1.7]

SEER: Surveillance, Epidemiology, and End Results; NCDB: National Cancer DataBase; OS: overall survival; RFS: relapse-free survival; CI: Confidence interval; HR: Hazard ratio.

**Table 4:** Summary of available studies evaluating treatment of metastatic LCNEC.

Author	Type of Study	*n*. of Patients	Treatment	Results
Rossi et al. (2005) [85]	Retrospective	83 LCNEC	Platinum–etoposide vs. other regimens	Best results with Platinum–etoposide ORR 29% (2 CR) mOS 51 m vs. 21 m
Fujiwara et al. (2007) [86]	Retrospective	22 LCNEC	Platinum-based or paclitaxel	Both irinotecan and paclitaxel may be active against LCNEC. mOS 10.3 m, 95% CI [5.8–14.8] vs. 10.3 m, 95% CI [0–21.8]
Sun et al. (2012) [13]	Retrospective	45 LCNEC	SCLC-based (11) vs. NSCLC-based (34)	SCLC-based therapy is more appropriate than an NSCLC-based one mOS for total population 11.1 m, 95% CI [8.4–13.9] mPFS 6.1 vs. 4.9 m (*p* = 0.41) mOS 16.5 vs. 9.2 m (*p* = 0.10)
Shimada et al. (2012) [75]	Retrospective	25 LCNEC vs. 180 SCLC	Platinum-based CT/CRT	Efficacy of chemotherapy and/or radiation therapy is similar between LCNEC and SCLC patients ORR 61 vs. 63% 1y OS 34 vs. 49%
Niho et al. (2013) [87]	Prospective (phase II, single arm)	30 LCNEC, 10 SCLC, 1 NSCLC	Cisplatin–irinotecan	Combination is active in LCNEC, but appears to be inferior compared to SCLC RR 46%, 95% CI [28.3–65.7%] vs. 80%, 95% CI [44.4–97.5%] mOS 12.6 m, 95% CI [9.3–16.0] vs. 17.3 m, 95% CI [11.2–23.3]
Le Treut et al. (2013) [81]	Prospective (phase II, single arm)	42 LCNEC	Cisplatin-etoposide	The outcomes are similar to those of SCLC mPFS 5.2 m, 95% CI [3.1–6.6] mOS 7.7 m, 95% CI [6.0–9.6]
Christopoulos et al. (2017) [88]	Prospective (phase II, single arm)	49 LCNEC	Carboplatin + Paclitaxel + everolimus	The combination is effective in first-line treatment ORR 45%, 95% CI [31–60%] DCR 74%, 95% CI [59–85%] mPFS 4.4 m, 95% CI [3.2–6] mOS 9.9 m, 95% CI [6.9–11.7]

mOS: median overall survival; mPFS: median progression-free survival; DCR: disease control rate; ORR: overall response rate; CR: complete response; CI: Confidence interval.

**Table 5:** Ongoing clinical trials in advanced LCNEC.

NCT	Phase	N	Tumors	Setting	Experimental Arm	Primary Endpoint	Status
NCT02834013 (DART SWOG 1609)	II	818	Rare tumors (including LCNEC)	Progressed during or after one line of chemotherapy	Arm 1: nivolumab + ipilimumab. Arm 2: nivolumab	ORR	Recruiting
NCT03976518 (CHANCE)	II	43	NSCLCs of rare histology	Progressed during or after at least one line of chemotherapy	Atezolizumab	DCR	Recruiting
NCT03728361	II	55	*Cohort 1*: SCLC; *Cohort 2*: Metastatic NEC of any grade/primary site (including LCNEC)	*Cohort 1:* progressed or recurred after platinum-based chemotherapy with immunotherapy; *Cohort 2*: Any line	Nivolumab + temozolomide	ORR	Active, not recruiting
Eudract 2020-005942-41 (DUPLE)	II	49	LCNEC	1st-line	Durvalumab + carboplatin + etoposide × 4 → durvalumab	1-year OS rate	Recruiting
NCT05126433 (EMERGE-201)	II	60	Advanced or metastatic solid tumors (including LCNEC)	Progressed on platinum-based regimen (irrespective of number of prior lines)	Lurbinectidin every 3 weeks	ORR	Recruiting
NCT03591731 (NIPINEC)	II	180	Poorly differentiated neuroendocrine tumors, including LCNEC	Progressed after one or two lines of treatment, including at least one line of platin-based chemotherapy	*Arm A*: Nivolumab *Arm B*: nivolumab + ipilimumab	ORR	Recruiting

N: planned number of patients; ORR, objective response rate; RR, response rate; DCR, disease control rate; NSCLC, non-small cell lung cancer; LCNEC, large cell neuroendocrine cancer; SCLC, small cell lung cancer; NEC, neuroendocrine carcinoma.
